# Trust and professionalism in science: medical codes as a model for scientific negligence?

**DOI:** 10.1186/s12910-021-00610-w

**Published:** 2021-04-14

**Authors:** Hugh Desmond, Kris Dierickx

**Affiliations:** 1 Centre for Biomedical Ethics and Law, KU Leuven, Leuven, Belgium; 2 Department of Philosophy, University of Antwerp, Antwerpen, Belgium

**Keywords:** Research integrity, Medical professionalism, Trust, Science policy, Negligence

## Abstract

**Background:**

Professional communities such as the medical community are acutely concerned with negligence: the category of misconduct where a professional does not live up to the standards expected of a professional of similar qualifications. Since science is currently strengthening its structures of self-regulation in parallel to the professions, this raises the question to what extent the scientific community is concerned with negligence, and if not, whether it should be. By means of comparative analysis of medical and scientific codes of conduct, we aim to highlight the role (or lack thereof) of negligence provisions in codes of conduct for scientists, and to discuss the normative consequences for future codes of conduct.

**Methods:**

We collected scientific and medical codes of conduct in a selection of OECD countries, and submitted each code of conduct to comparative textual analysis.

**Results:**

Negligence is invariably listed as an infraction of the norms of integrity in medical codes of conduct, but only rarely so in the scientific codes. When the latter list negligence, they typically do not provide any detail on the meaning of ‘negligence’.

**Discussion:**

Unlike codes of conduct for professionals, current codes of conduct for scientists are largely silent on the issue of negligence, or explicitly exclude negligence as a type of misconduct. In the few cases where negligence is stipulated to constitute misconduct, no responsibilities are identified that would help prevent negligence. While we caution against unreasonable negligence provisions as well as disproportionate sanctioning systems, we do argue that negligence provisions are crucial for justified trust in the scientific community, and hence that there is a very strong rationale for including negligence provisions in codes of conduct.

**Supplementary Information:**

The online version contains supplementary material available at 10.1186/s12910-021-00610-w.

## Background

Science has long left its amateur and gentleman-scientist past behind [1], yet it is only relatively recently, in the past two decades, that the scientific community has started strengthening its structures of professional self-regulation. Codes of conduct have been introduced; researchers are increasingly given research integrity training; and integrity commissions are being set up to deal with transgressions of the codes of conduct [2, 3]. However, as will be documented in this paper, professional communities such as the medical community are acutely concerned with negligence: the category of misconduct where a professional does not live up to the standards expected of a professional of similar qualifications. It is unclear to what extent the scientific community is or should be concerned with negligence. Despite its importance for professional codes of conduct, the category of negligence is rarely mentioned and has not received systematic analysis in the literature on the definitions of research misconduct [4–7].

What is negligence? For instance, if a construction company fails to meet all the latest safety guidelines, and one of its employees suffers a workplace accident, then the company can potentially be held liable—not because it knowingly put the employee in danger, but because it did not do everything reasonably expected of it to safeguard employees' safety (i.e., remain abreast of latest guidelines). In general, negligent transgressions are those that do not involve a conscious intention (or even a conscious disregard of the risks), but a failure to take reasonable precautions.^1^

Negligence is interesting for philosophers of law and jurisprudence scholars because it challenges the traditional voluntaristic way of conceiving culpability, because an agent is not only held responsible for actions under their *actual* control [9], but also for actions which *would have been* under their control if they had behaved as expected of “a reasonably prudent person” [8]. However, the general implications of negligence for moral culpability [2, 3] or legal culpability [12] fall outside the concern of this paper. As will be documented in this paper, negligence plays a crucial role in justifying trust in professionals, and hence the purpose here will be to draw lessons for research integrity and scientific professionalism.

In particular, this paper will focus on the functional role that negligence plays in professional codes of conduct (“professional codes”), and what role it should play in codes of conduct for scientists (“scientific codes”). Two questions in particular will structure the enquiry:

1. to what extent are codes of conduct for scientists currently concerned with negligence, compared to professional codes of conduct?
2. what negligence provisions should be included in codes of conduct for scientists?

The first question concerns the empirical state of affairs of how codes of conduct are currently constructed, and in this paper it is tackled by analysing codes of conduct for physicians and for scientists in nine OECD countries. Codes of conduct for physicians are chosen since sociologists often view the medical community as a paradigm of a professional community [13]. The second question is the normative question, and we will make positive recommendations for how negligence provisions should be included. However, before we turn to these questions, first it is necessary to give some additional background: why are negligence provisions so important for the professions, and why does this provide a *prima facie* rationale for the science community to pay greater heed to negligence provisions?

## The rationale for negligence provisions

In this background section we wish to argue in more detail why negligence provisions matter: they fulfil a crucial role for the principles of autonomy and trustworthiness that characterize the *logic* *of professionalism* [13]. We will first sketch this rationale for professional negligence provisions in general, and then for scientific negligence provisions in particular.

### The rationale for professional negligence provisions

The logic of professionalism is an approach towards organizing work where the practitioner and the community of practitioners are given a large degree of *autonomy* to carry out the work as they see fit [13].^2^ Professionalism is an ideal-type to which real occupations partake to varying degrees, and typically two rival logics are mixed in as well: the logic of bureaucracy, where managers control  how the work is carried out, and the logic of markets, where clients or customers control how the work is carried out. Sociologists have often taken medicine to be a paradigmatic example of an activity organized according to professionalism in the sense that there is a large degree of operational autonomy [14, 15]; however, it is clear that also physicians operate under some bureaucratic control (e.g. audits) as well as market incentives (and increasingly so in the previous decades: [16]).

In the logic of professionalism, professional *autonomy* and *trust* in professionals are two sides of the same coin. Trust in professionals means that relatively few demands for control and transparency made of professionals (and insofar as such demands are made, they can be interpreted as reflecting an erosion of trust; see also [17]). Hence trust is what allows professionals to maintain their autonomy. Conversely, professionals’ autonomous decision-making does not mean that the decision-making is voluntaristic, but rather that it is guided by what Freidson called an ideal of service [13]. For instance, physicians and medical professionals are typically oriented to the ideal of care, and this implies, inter alia, that in their decision-making they prioritize care for the patient over following self-serving incentives. In this way, because professional autonomy is grounded on a service ideal, both patients/clients and wider society are justified in trusting autonomous professionals. This trust, in turn, allows professionals to maintain their autonomy.

This fundamental connection between trust and professional autonomy is crucial for understanding the key role that negligence provisions play in well-designed professional codes of conduct. As indicated in the introduction, accusing someone of “negligence” presupposes that a certain standard of prudence was expected from that person. The person is culpable because they *should have known* about the possibility of the bad outcome. In daily life, adults can be held responsible because of negligence simply because they did not act according to common sense standards of prudence (think of, e.g., the category of negligent homicide [8]). Finding someone to be negligently culpable is typically the least serious form of culpability (after ‘purposely’, ‘knowingly’, and ‘recklessly’), and is the only form of intent where the undesirable outcome was not known as a possible outcome by the transgressor (for more discussion, see [9]).

Negligence provisions are especially important for the logic of professionalism because the level of prudence expected of a professional—at least with regard to their professional activity—is higher than that of a layperson. Substantial trust is placed in (and autonomy is granted to) a professional, and this trust goes beyond simply trusting their good intentions: trust is also placed in their knowledge and competence [19]. Any client or patient who calls on the services of a professional is placed in a vulnerable position, and when the professional does not live up to professional standards, then this is a sign that trust in this particular professional was misplaced. In other words, negligent action by a professional is tantamount to a breach of the client’s or patient’s trust.

Hence the importance of categorizing negligence as a type of transgression in professional codes of conduct: this is an explicit commitment by the professional body that clients *should by default* place trust in professionals, because when this trust turns out to misplaced because of carelessness or imprudence on behalf of the professional, then the client is not at fault, for instance for being naïve. On the contrary, the professional is culpable for failing to live up to that default trust.

Just to emphasize this point: imagine if a professional body would *not* recognize negligence as a transgression. How could clients make the decision whether or not to trust a professional? The onus would then be on the client to do the necessary due diligence in order to decide whether or not to place trust. Does the professional have sufficient competence? Does the professional have the best interests of the client at heart? If the professional cannot be held culpable for professional negligence, then the principle of *caveat emptor* would apply for the prospective patient or client. However, how can a patient evaluate the competence of, for instance, a heart surgeon? Not only are the services offered by professionals often urgent and essential, but *by assumption* patients or clients lack the requisite knowledge and training they would need in order to evaluate how competent a given professional is. (If substantial knowledge and training were not necessary, there would be no need to professionalize the activity in the first place: see [13].) “Buyer beware” can work for purchasing second hand goods, but it cannot for medical services or other important and highly technical professional services.

In sum, giving (medical) professionals discretion without holding them responsible for negligence incoherently gives them rights without holding them to the naturally associated responsibilities. Or in other words: if negligence is not problematized by professional bodies, this implies nothing less than the collapse of the logic of professionalism.

### The rationale for scientific negligence provisions

Should the activity of scientific research adhere to a logic of professionalism? Elsewhere it has been argued that it in fact should [2], and the crux of Desmond’s argument was to point to the unavoidable role played by individual judgment in the activity of scientific research (see also [20, 21]). The day-to-day activities of formulating hypotheses and developing methodologies, overcoming challenges, analysing and interpreting data: in all of these, scientists have a large degree of operational autonomy whereby they cannot simply follow formulaic methodologies but must use their individual discretion. Moreover, many other scientific activities that support the scientific community—refereeing, supervising, editing—involve scientists following their individual judgment rather than a set of rules (as would be in a logic of bureaucracy).

This type of individual autonomy is not just desirable for scientific research; it is also inevitable given the highly technical nature of scientific knowledge. Sociologists speak of the knowledge underlying professional activity being “esoteric”, by which they mean that it is inaccessible without education and training [13]. And science plays a crucial role in developing the knowledge of the professions—to such an extent that some have argued that professions simply *are* science-based occupations [22]. In any case, this line of reasoning obviously also applies the “occupation” of doing scientific research. If any body of professional knowledge could qualify as “esoteric”, then it would be the body of knowledge necessary for a scientist to do their research. In fact, a given research project may be so specialized that may be difficult even for scientific peers to fully understand it, let alone non-scientists. In this sense, the degree of autonomy of scientist is an inevitable consequence of the specialized, esoteric knowledge required for doing scientific research.

In sum, an important dimension of the logic of professionalism, namely individual autonomy, is part and parcel of scientific research. Note, however, that this does not imply that individual autonomy is absolute and that the decision-making of individual scientists cannot be influenced by non-scientific factors. This is not the case: the preferences of granting agencies, private companies, and governments may influence scientific decision-making (e.g., in choice of research topic).^3^ The lesson here is that individual autonomy may be curtailed—and the logic of professionalism may thus be mixed in with other organizational logics—but it cannot be eliminated since scientific research necessarily depends on individual scientists retaining a large degree of operational autonomy.

However, even if a lot of elements are in place for considering scientific research to be a professional activity, there are challenges involved. One challenge is that it is not immediate clear what “scientific negligence” could mean. *How* scientific negligence should be understood is exactly the purpose of this article, and will be addressed in later sections. For the moment, however, we would like to make the case *that* there should be a category of scientific negligence. If negligence is not explicitly understood as a transgression of integrity norms, then trust in scientists is undermined. To show this, we disambiguate individual trustworthiness from collective trustworthiness.

#### Individual trustworthiness

Consider the following scenario. Assume that scientist A claims to know $\varphi$, for instance, some claim about the degree of climate change. Under what conditions can a non-scientist B defaultly trust A? One condition for trust is that B should be able to assume that A is not *negligently* claiming $\varphi$, i.e., that A has taken all reasonable precaution to avoid being wrong. If A were not to take reasonable precautions to avoid error, and moreover, if it were common knowledge that scientists do not take such reasonable precautions, then default trust in A’s assertions would not be justified and B could be considered as naïve if they were to trust without any due diligence.

The issue of negligence comes especially to the fore in connection to expert communication, such as the communications concerning climate change or COVID-19. These communication can be considered as crucial services a scientist provides to the community [24]. Moreover, in such cases, A’s claim $\varphi$ may have far-reaching impact on social norms and thus B’s life. The non-scientist B is placed in a vulnerable position, and this increases the need for B to be able to trust A, and thus to be able to assume that A has taken reasonable precautions to avoid negligence. However, note that this does not mean that B should have to assume that A has avoided error. Error as such is inherent to any scientific activity, including expert communication, and error need not imply a lack of trustworthiness. Only *negligent* error can play that role: a negligent error is one that a scientist could have avoided if he or she had taken the reasonable precautions that are expected of all scientists of comparable training.

In sum, given the inevitable autonomy with which research (and supporting activities) must be conducted, non-specialists often cannot evaluate scientific claims for themselves, but must make the decision to trust or not to trust. If the scientists do not take all reasonable precautions to avoid error, then trust in their pronouncements would not be justified. Hence safeguarding individual trustworthiness is the first reason why it is important to include a clear stance on negligence in scientific codes of conduct.

#### Collective trustworthiness

One could respond that collective trustworthiness is more important than individual trustworthiness. After all, it would be reasonable to judge the claim of an individual scientist to be trustworthy only when it has been vetted by peer-review. While of course peer-review has its own discontents, it does illustrate how the trustworthiness of individual scientists is bound up with the trustworthiness of the scientific community as a whole. While the former depends explicitly on intentions and competences, the latter depends on the *value norms* and *standards of competence* of the community.

It may seem strange to claim that, despite the flux characterizing any scientific state of the art, the scientific community needs clear standards of competence. However, even if such standards are not always formulated explicitly, they are often implicit. For instance, the practise of peer-review presupposes the existence of methodological standards. Moreover, having such standards is a precondition to being able to identify what precautions can be “reasonably expected” of a scientist, and hence to be able to distinguish between an “honest error” and “negligent error” in a principled manner. Thus, without standards of competence that can only be defined at the level of the community, trust in scientists cannot be placed defaultly.

The importance of collective value norms is also crucial for collective trustworthiness. For instance, in “perverse” research cultures, transgressions of integrity norms can be normalized. Examples include the exaggeration of research findings and authorship practises [25]: these are questionable research practises (QRP) that yet do not constitute flagrant transgressions (FFP). When there are recognized integrity norms, then engaging in a QRP that is normalized in a subcommunity would constitute a category of negligence. This is important for collective trustworthiness, because the discovery of perverse research cultures by the broader public would undermine the justifiability of public trust in science.

How do individual and collective trustworthiness relate? When there are explicit standards of competence and value norms, then the trustworthiness of individuals and that of the community can become dissociated to a certain extent. If an individual commits some kind of negligent misconduct, then this need only reflect on the trustworthiness of the individual, since they are failing to live up to collective standards and norms. By contrast, when collective standards and norms are absent or not recognized, then the community can bear responsibility for individual misconduct. For instance, if an individual scientist publishes a fraudulent study, then a certain degree of culpability could be attributed to the peer reviewers and editors involved, if they did not have appropriate standards or norms in place.

In sum, negligence norms enhance the scope of individual and collective responsibility, and include taking all reasonable precautions for delivering competent and trustworthy research. By contrast, if negligence would not be problematized by the scientific community, then trust in assertions by scientists may not be entirely *un*justified, but the type of default trust implied by the logic of professionalism would not be justified. This, in turn, would motivate further curtailments of scientific autonomy by the public. Hence the rationale for explicitly understanding scientific negligence as a form of misconduct in codes of conduct is to help safeguard justified trust in scientists and the scientific community.

## Methods^4^

To what extent do current scientific codes of conduct include negligence provisions? To map this issue, we collected national codes of conduct in science and medicine for eight countries (USA, AU, CA, UK, BE, FR, DE, IT) plus any relevant Europe-wide code of conduct. The search was carried out between February and May 2019, and updated in October 2020. The primary sources of information for the scientific codes of conduct were the websites of national research councils, national agencies on research integrity, national scientific fund, or national academies of science. Results were cross-checked with an internet search engine (Google) with search terms ((“research integrity” OR “scientific integrity” OR “science integrity”) AND “ <name of country> ”). Only codes of conduct or codes of ethics were included. Other documents relevant to how science and the professions are practiced were excluded, e.g., legal documents not strictly pertaining to regulation of professions or sciences (e.g., in copyright law, tort law, or privacy laws) and documents regulating scientific research on human and animal test subjects. In the cases where there were multiple national-level codes of conduct, we followed the methodology in [3] to select the “leading” document, which refers to the document that is considered central and authoritative within the relevant national context. For the medical codes of conduct, primary sources of information were national medical associations whose boards confer licensing or registration to physicians. Due to the centralized and unified nature of the medical profession, there was no reasonable ambiguity involved in choosing a code of conduct. In some countries there are minority professional organizations (e.g. Association of American Physicians and Surgeon in the USA), which were not accounted for in this study. However, since the purposes here are not to conduct a systematic review, this limitation in methodology was deemed not to affect the conclusions drawn from the results. All details concerning the selected documents are provided in the supplementary materials.

Does focusing on the medical profession (instead of professions more generally) limit the scope of conclusions? Not necessarily, because, as already noted, the medical profession is often viewed by sociologists as a paradigmatic instantiation of professionalism. So any systematic difference found between scientific and medical codes of conduct has bearing on the general question to what extent the scientific community currently is adopting the logic of professionalism.

Each code of conduct was submitted to textual analysis to identify passages pertaining to negligence. For this it was necessary to operationalize of the concept of negligence. The previous section sketched the general rationale for negligence provisions; however, there are several concrete ways in which professionals can act to avoid negligence. In general, negligence is avoided by professionals having awareness of their competences and of the standards expected of them. So, whenever possible, the professional must take all reasonable measures to assure they can act according to accepted standards of competence: hence, for instance, the importance to keep abreast of developments in the field, or to undergo additional training or education. If this is not possible, then the response to a lack of requisite competence is either to refrain from undertaking services, and to refer to client or patient to a colleague.

Given this reasoning, we judged a code to view “negligence” as a violation of the norms of integrity if there was *at least one* of the following:

- A provision emphasizing the responsibility for providing *competent* services only.
- A provision designating a responsibility to recognize absent competence in oneself: if lacking requisite competence, a practitioner must not carry out the work, and refer to a colleague who does possess the requisite competence.
- A provision designating a responsibility for maintaining competence: a practitioner has the responsibility for keeping up-to-date on the standards in their field.
- A provision designating a general responsibility to uphold “standards of professionalism”, which are assumed to include standards of competence.

Thus, for instance, the UK “Good Medical Practice” code stipulates that physicians must be competent in all aspects of their work (principle 7); keep their professional knowledge and skills up to date (principle 8); and work within the limits of their competence (principle 14). Similarly, albeit in different wording, the “Professional Code for Physicians in Germany” stipulates that physicians must possess the requisite qualifications and remain up to date on the medical state of the art (article 2); they also must maintain and develop their competences by continuing medical training (article 4). Thus, a single code of conduct may include multiple negligence provisions.

## Results

We found a striking difference between codes of conduct in the two communities (Table 1): while negligence is invariably listed as an infraction of the norms of integrity in codes of conduct for physicians, it is rarely so in codes of conduct for scientists. Moreover, in the few cases where negligence is listed as an infraction of a scientific code, no detail is provided on what precise responsibilities scientists have for avoiding negligence: this raises questions on how such negligence provisions should actually translate into conduct.

**Table 1:** Is negligence listed as an infringement of the code of ethics? Scientific versus medical codes of conduct. Y = yes; N = no; / = code not available. For all relevant passages, see supplementary materials

Country	Code of Conduct	
	Scientific	Medical
USA	N	Y
Canada	N	Y
Australia	Y	Y
UK	Y	Y
Europe	N	/
Belgium	N	Y
France	N	Y
Germany	Y	Y
Italy	N	Y
Netherlands	Y	Y

All examined medical codes of conduct contain provisions that, according to the operationalization of the concept of negligence above, can be judged to be provisions that explicitly proscribe negligent behavior. In other words, negligence is an infringement of medical codes of conduct, regardless of national context. Other studies have indicated a focus on competence (first condition for negligence) in codes of other professions, such as those for legal professionals or psychologists [2], so the widespread inclusion of negligence provisions in medical codes of conduct is unsurprising.

By contrast, most of the examined codes of conduct for scientists either explicitly exclude negligence as a category of infringement, or are silent on the issue. The former is the case, for instance, for the Federal Research Misconduct Policy, which stipulates that “a finding of research misconduct requires that: […] the misconduct be committed intentionally, or knowingly, or recklessly.” The Federal Policy also limits the definition of research misconduct to falsification, fabrication, or plagiarism, or FFP (OSTP 2000, p. 76262), which are in fact the categories of misconduct most likely to be committed intentionally, knowingly, or recklessly.

Some codes of conduct do explicitly mention the possibility of negligent misconduct. For instance, the UK “Concordat to Support Research Integrity” includes “gross negligence” as a potential form of misconduct in misrepresenting data or when the duty for care for human test subjects is breached. However, these negligence provisions concern only specific types of behavior, and thus do not apply to negligent infringements of any integrity norms (for instance, regarding authorship norms). In Sect. 4 we argue why this is problematic.

Finally, some codes, like the DFG “Guidelines for protection good scientific practice” and “Netherlands Code of Conduct for Research Integrity” do make a general provision regarding “gross negligence”:

“Only deliberate or grossly negligent infringements defined in a set of regulations are considered scientific misconduct.” (DFG 2019)

“When it amounts to gross negligence, a questionable research practice or ‘sloppy science’ is more than a matter of mere error or carelessness but rather something that can undermine the very integrity of research.” (KNAW 2018)

However, the category of “gross negligence” is ambiguous: sometimes it is understood as negligence, but other times as recklessness. Black’s Law Dictionary notes this polysemy, and distinguishes between a first meaning of gross negligence as “a lack of slight diligence or care” and a second meaning, “a conscious, voluntary act or omission in reckless disregard of a legal duty” (i.e., recklessness) [8]. In the context of prosecutions of medical misconduct, the first meaning of gross negligence is the more common one [26]. The DFG and KNAW codes, as written, do not offer resources to disentangle these two meanings of gross negligence. For instance, the Netherlands Code of Conduct for Research Integrity, contrasts “gross negligence” with “carelessness or ignorance”^5^: this is confusing since “recklessness” always involves carelessness and “negligence” may either involve carelessness or simple ignorance. Hence this contrast does not allow one to infer what precisely the understanding of “gross negligence” is (i.e., as negligence or as recklessness) in the Netherlands Code of Conduct for Research Integrity.

The only examined code where negligent infractions of the code are included as a category of misconduct is the Australian Code for the Responsible Conduct of Research. Even though the code does not offer any precise definition of scientific negligence, nor any detail on how scientists should act in order to avoid negligence, we conclude that this code is the only one of the ten examined where a general negligence provision, pertaining to all types of infringement of integrity norms, is included.

From this brief, non-exhaustive overview, one can infer (1) scientific negligence is often not stipulated as a type of transgression of integrity norms, and (2) when it is, the meaning of negligence is often left unclear, and without exception (at least, for the codes reviewed) the concomitant responsibilities for avoiding negligent behavior are not stipulated.

## Discussion: principles for negligence provisions

How should the absence of negligence provisions in scientific codes of conduct be evaluated? One could accept the general rationale for negligence provisions, namely to *justify trust* in the scientific community, and still reject that they *should* be included in scientific codes of conduct. For instance, one could hold that such provisions would be very difficult to enforce. One could also hold that providing rules on sanctioning is not the primary purpose of a code of conduct, but that it is rather meant as an ethical guide for scientists in their work (see also Sect. 4.3 in [3]). In this discussion section we sketch how negligence provisions should be designed: how standards of competence should be approached, as well as how to avoid pitfalls in overemphasizing enforcement and in defining negligence too broadly.

### Standards of competence

A finding of negligence means that a mistake was made even though a “reasonable precaution” could have avoided that mistake. Such “reasonable precautions” are defined relative to the standards of competence that can be expected of similarly certified professionals (or scientists). Hence, stipulating responsibilities to avoid negligence is only possible if there are also standards of competence to which the work of the professional or scientist can be held.

One could be tempted to reject any attempt to stipulate “standards of competence” for scientific research. After all, a lot of analytic philosophy of science in the twentieth century searched for a universally applicable and valid methodology (falsification, confirmation, etc.)—a methodology that moreover was to distinguish legitimate scientists from their pseudo-scientific rivals [27]. These efforts failed, and while one need not go so far, as some did, as to reject all scientific methodology and hence standards of competence [28], one could remain skeptical of efforts to include general standards of competence in a code of conduct meant for scientists across all domains.

Nonetheless, similar remarks could be made of professional standards: different specializations have different standards, and many such standards may not be codified with precision or may even be tacit. Yet that has not prevented negligence provisions from being included in professional codes of conduct. Negligence provisions communicate the message that, whatever the standards are within a specialization, the practitioner has the responsibility to know and follow them. 

Similarly, while one must surely grant that there is no universally applicable standard scientific methodology, this does not mean one could not identify scientific standards of competence that are more limited in scope. In fact, as previously noted, such standards are in fact implicitly invoked in the process of peer review: without such standards, submitted work could not be judged to be of “good quality” or “poor quality”. One could plausibly surmise that standards of competence in fact are tacitly known by practitioners of a sub-field: each sub-field has particular expectations on what a well-written or well-constructed article looks like, what the appropriate level of specificity of the argumentation should be (if relevant), how the data are to be represented (if relevant), what types of literature should be engaged with, and so on.

One question facing authors of codes of conduct is thus *how precise* to make negligence provisions. Precisely defined provisions, based on explicit standards of competence, would allow for verification, i.e., to answer the question: does a certain action constitute negligence or not? By contrast, a vaguely defined provision remains ambiguous, would not allow for findings of negligence, and thus would be unenforceable. As an illustration, consider a competence like “critical thinking”. This is an example *par excellence* of a competence that would be difficult or impossible to define by means of verifiable criteria. This would mean that third parties could never verify whether a particular scientist or project lived up to the relevant standards of critical thinking. The prospect of setting up an investigation to examine whether some scientist respected standards of critical thinking is, of course, absurd; the manifest absurdity reflects how some scientific ideals remain aspirational and non-enforceable. Yet non-enforceability does not mean it should not or cannot be affirmed as an important standard. We come back to this in the next subsection in connection to the tension between the ethical function of negligence provisions and their legal function (enforcement).

Yet some standards lend themselves to explicit definition, especially those concerning *procedures.* Actions whereby such procedures were not followed, whether by conscious intention or not, would then be culpable as negligence. Examples of such procedural negligence include:

1. Making easily avoidable errors when handling data, such as mislabeling samples.
2. Publishing without first trying to replicate one’s results when such replications are relatively easy to do.
3. Carrying out statistical analysis despite not having the requisite competences, with the danger of wrong statistics being applied, P-values not being interpreted correctly, etc.

Many of the support activities in the scientific community also follow relatively clear standards of competence. Institutions have responsibilities, and when these are not met, institutions may be found to have acted negligently. Individual researchers acting in capacities of referee, editor, or supervisor also must follow relatively definable standards, with associated negligences. For instance, a referee may review an article or proposal negligently when, for instance: insufficient time or energy is devoted to the task, thus not taking reasonable precautions to avoid forming egregiously wrong judgments of the article or proposal; not refusing to review the article or proposal if lacking in competence. An editor *may* neglect their responsibilities by, for instance: accepting peer-reviews by scientists who clearly lack the necessary competences; asking peer-reviewers to make editorial judgments; not specifying reasonably clear standards or expectations for referees. Finally, a supervisor may be negligently culpable if, for instance: they do not take reasonable precautions to avoid members of the research team from taking large risks; or if they do not offer sufficient guidance to supervisees.^6^

In sum, the elucidation of standards of competence may not be easy or even always possible. However, this does not preclude affirming the importance of negligence, and moreover, it seems possible to define standards of competence for following scientific procedures as well as for support activities such as acting as referee, editor, or supervisor. Formulating these standards, and integrating them into codes of conduct would play an important role in emphasizing the importance of avoiding negligence.

### Pitfalls and unintended consequences

We would like to close the discussion with addressing some of the dangers of negligence provisions. Negligence broadens the scope of culpability of scientists, and introducing negligence as a norm may produce unintended consequences if not done with prudence. Inappropriate standards of competence, where unwitting scientists would be found guilty of misconduct in harsh and inappropriate ways, could have a similar “chilling effect” that criminalizing even egregious research misconduct would likely have [29]. After all, when faced with increased personal risk due to negligence provisions, scientists may simply decide to not to expose themselves to risk and to not undertake certain activities which are nonetheless crucial for the scientific enterprise. Alternatively, a scientist may respond with heightened distrust, taking inappropriate measures to control outcomes and to minimize error. We discuss each in turn.

#### Diminishment of scientific activity

It is important to emphasize that the function of codes of conduct is primarily to provide clarity about the ethical principles underlying scientific activities, and only secondarily (if at all) to give detail about the sanctioning system attached to integrity transgressions. A sanctioning system is constrained by a whole host of other ethical and legal principles, such as the principle of proportionality. Hence, even if an act of negligent research could be considered a transgression against the norms of integrity, great care would be needed in choosing the appropriate response to that transgression, to avoid doing even greater damage to the scientific enterprise. 

Here is an example: if a researcher (likely) exaggerated their research findings, this does not mean that a formal institutional procedure regarding negligence should be started. Even the starting of such procedures is harsh: they are fraught with uncertainty, often involve reputation-loss for the accused, can lead to an erosion of trust in the research partners of the accused scientist (who blew the whistle?), and can erode collegial relations within research institutions. Moreover, the line between sensationalizing claims and making them engaging and digestible (i.e., “selling”) is a fine one. A overly harsh sanction could have the unintended effect of discouraging future scientific activity.

The sanctions would not even need to be harsh for negligence provisions to have a chilling effect. For instance, if referees or editors were under the impression that they would need to go to an unreasonable degree of scrupulousness (at the pain of being found culpable of negligence), they may decide that taking up reviewing or editing duties is simply not worth the risk or effort. In this way, inappropriate negligence provisions can express a distrust towards individual scientists, who then respond by pulling back.

One positive suggestion in this regard would be to embed principles about negligence in what in professional contexts is known as “just culture”, which is often described as an organizational culture where the guiding response to mistakes is not “who is to blame” but “what went wrong” [30, 31]. It is clear that, if many QRPs would be classified as (negligent) misconduct, then the scientific community would need to avoid a blame culture and take all reasonable steps to strengthen a just culture. Developing such a just culture would also entail moving away from the emphasis on FFP in many scientific codes of conduct, since these almost always involve conscious intentions (and are thus concern “who is to blame”).

A sanctioning system such as that within a just culture—where sanctions are aimed at behaviors rather than persons—is thus one candidate for how to include negligence provisions while avoiding a diminishment of scientific activity. In a just culture, the sanctioning of negligence does not suppress individual scientific agency and thus does not diminish the ethical function of integrity norms. 

#### Negligence provisions and distrust

The rationale for negligence provisions is to safeguard justified trust in a scientific or professional community; however, ironically, if *too* much precaution is expected, this may have the perverse effect of increasing distrust.

As an illustration, consider a situation where scientific institutions can be held responsible for negligent conduct of an individual member of the institution. Then such institutions, fearful of prosecution in courts of law, would have an incentive to exact greater degrees of bureaucratic control of individual scientists. This would be an example where too much responsibility is expected of institutions, and that this has the perverse effect of curtailing individual autonomy.

Another, similar example would be a negligence provision where supervisors would be expected to double check the work of lab members. Thus, if a lab member were found to be guily of misconduct, then the supervisor, if they had not double checked the lab member’s work, could be considered negligently culpable. In such an environment, supervisors would be incentivized to exert a very high level of control on lab members, with much wasted time and energy.

It is also crucial for the effectiveness of RI training that negligence provisions be reasonable. Such training, at least when effective, establishes integrity norms as intersubjective social norms: I know the norm, you know that I know the norm, I know that you know that I know the norm, and so on. By coordinating expectations in this way, setting norms—even in a code of conduct—can impact patterns of behavior even without explicit sanctioning systems. Yet, if the specified norms are unreasonable, and if it becomes intersubjective knowledge that they are unreasonable, then integrity norms lose all their normative force and become mere window dressing, divorced from actual research culture.

In sum, great care should be made when adding negligence provisions to make sure that the associated expectations are *reasonable,* and in particular that setting such standards would not have detrimental effects on trusting relationships between colleagues and that they instead can foster a healthy and flourishing research culture.

## Conclusions

Scientific negligence should be problematized much more than it currently is in codes of conduct for scientists. Problematizing negligence is part and parcel of the logic of professionalism, and is key for keeping and deepening the trust of the wider society in the scientific community. In fact, if the scientific community is to genuinely adopt professionalism as an organizational structure, it must find a way to include negligence provisions in codes of conduct. Without these provisions, the rationale for trust in the scientific community and for scientific autonomy cannot be justified.

Yet at the same time, it is not easy to discern what precisely should be the path towards including negligence provisions. For some scientific activities—following procedures, or support activities such as reviewing—it may be relatively easy to identify standards compared to the core activity of innovative research. Nonetheless, even then there are considerable pitfalls, because, if done poorly, negligence provisions can lead to a drive towards control, which in turn can damage trust between scientific agents (both individuals and institutions). In this article we discussed pitfalls regarding the sanctioning system and expectations of precaution. In sum, negligence provisions should be included in scientific codes of conduct, but it is paramount this should be done in a reasonable way, as a means to enhance rather than diminish trust between scientists.

## Supplementary Information

**Additional file 1:** The supplementary materials give additional information on: (1) the search and selection of the analyzed documents, (2) all identifying data of the documents, and (3) what precise negligence provisions were identified.

## Data Availability

All consulted documents are available in the supplementary materials.

## Abbreviations

FFP: Fabrication, falsification, plagiarism
QRP: Questionable research practises
RI: Research Integrity

## Footnotes

1 This understanding is continuous with the definition of negligence in Black’s Law Dictionary, “The failure to exercise the standard of care that a reasonably prudent person would have exercised in a similar situation …”[8]

2 It is important to emphasize that “professionalism” is thus a technical concept, and should be distinguished from common uses of the word “professional”, for instance: as being remunerated for a type of work (“they were successful and subsequently did the activity professionally”), or as a synonym for doing quality work (“they did a very good job, very professional”). The logic of professionalism is what underlies these more common-place uses of the word “professional”.

3 Note that the research funded by government and by private companies represents two thirds of research in the United States by amount of funding [23].

4 Methodology and results are based on, but further develop previous and related work on competence provisions in codes of conduct; see Author 1 2020.

5 See p. 24: “The level to which non-compliance was intentional and whether it was a form of gross negligence or was the result of carelessness or ignorance” (p. 24).

6 See the ICMJE (International Committee for Journal Editors) or COPE (Committee on Publication Ethics) for further examples of standards expected from scientists acting in these roles.
