# Supplementary material for: Trust and professionalism in science: medical codes as a model for scientific negligence?
Source: BMC Med Ethics. 2021 Apr 14;22:45. doi: 10.1186/s12910-021-00610-w (PMC8046265; doi:10.1186/s12910-021-00610-w)
Supplement: Supplementary file 1 — Additional file 1: The supplementary materials give additional information on: (1) the search and selection of the analyzed documents, (2) all identifying data of the documents, and (3) what precise negligence provisions were identified. [file 12910_2021_610_MOESM1_ESM.docx]

**Supplementary Materials**

**Data Sources:** Documents with major position statements on service ideal and autonomy (often codes of conduct, but also laws, guidelines, or other policy documents) in science, medicine, law, and individual scientific disciplines (sociology, psychology, physics, and philosophy), and for the European Union plus nine countries (USA, AU, CA, UK, BE, FR, DE, IT, NL) were collected. The search was carried out between May and July 2018, and updated in October 2020. The primary source of information were the websites of national associations of scientific disciplines, national associations of professions, scientific funding bodies, and national research integrity organizations. All URLs to the codes of conduct (and issuing associations) are provided below.

**Data Selection:**  Only documents pertaining to research integrity or professional integrity were included. Other documents relevant to how science and the professions are practiced were excluded, e.g., legal documents not strictly pertaining to regulation of professions or sciences (e.g., in copyright law, tort law, or privacy laws) and documents regulating scientific research on human and animal test subjects. The review was limited to nine OECD countries plus the EU, and while this yields a small sample size (N=10), it is sufficiently well-balanced to allow for generalizations, since the countries considered play a leading role in formulating position statements concerning incompetence, autonomy and the service ideal in both science and the liberal professions. Selection was made of countries from both common law and civil law systems as these have different institutional traditions.

**List of Codes of Conduct**

Passages pertaining to service ideal are at the beginning of the codes of conduct. Relevant passages pertaining to competence and autonomy are noted.

1. **Science**
   1. **USA**

Title: Federal Research Misconduct Policy

Author: Office of Science and Technology Policy (OSTP)

Date: December 6, 2000

URL: https://www.govinfo.gov/content/pkg/FR-2000-12-06/pdf/00-30852.pdf

Relevant passage on negligent incompetence: “A finding of research misconduct requires that: (…) The misconduct be committed intentionally, or knowingly, or recklessly”

- 1. **Canada**

Title: NRC Research Integrity Policy

Author: National Research Council Canada

Date: October 15, 2013

URL: https://www.nrc-cnrc.gc.ca/obj/doc/about-apropos/policies-politiques/research_integrity-integrite_recherche/research_integrity.pdf

Relevant passage on negligent incompetence: Negligence is explicitly excluded as a violation of the code of conduct:

“Research misconduct does not include unintended error” (p. 7), and later “Each statement of findings must (…) identify whether the research misconduct was (…) committed intentionally, knowingly, or recklessly” (p. 27)

- 1. **Australia**

Title: Australian Code for the Responsible Conduct of Research

Authors: Australian Research Council and Universities Australia

Date: 2018

URL: <https://www.nhmrc.gov.au/sites/default/files/documents/attachments/grant%20documents/The-australian-code-for-the-responsible-conduct-of-research-2018.pdf>

Relevant passage on negligent incompetence: “Research misconduct: A serious breach of the Code which is also intentional or reckless or negligent.” (p. 5)

- 1. **UK**

Title: The Concordat to Support Research Integrity

Date: October 2019

Author: Universities UK

URL: https://www.universitiesuk.ac.uk/policy-and-analysis/reports/Documents/2019/the-concordat-to-support-research-integrity.pdf

Relevant passage on negligence: commitment 4

“breach of duty of care for humans involved in research whether deliberately, recklessly or by gross negligence, including failure to obtain appropriate informed consent”

And “…by gross negligence presenting a flawed interpretation of data”

- 1. **Europe**

Title: The European Code of Conduct for Research Integrity

Authors: European Science Foundation, All European Academies

Date: 2017

URL:

<https://ec.europa.eu/research/participants/data/ref/h2020/other/hi/h2020-ethics_code-of-conduct_en.pdf>

Relevant passage on Negligence: section 3.1, where “direct violations” of the code of conduct are limited to FFP (which involve conscious intention: see section 7.4 in main text).

- 1. **Belgium**

Title: Code of Ethics for Scientific Research in Belgium

Authors: Royal Flemish Academy of Belgium for Science and the Arts, The Royal Academy of Science, Letters and Fine Arts of Belgium

Date: 2009

URL: <http://www.belspo.be/belspo/organisation/publ/eth_code_nl.stm>

Relevant passage on negligence: no passage is dedicated to specifying extent of responsibility of individual researchers. In passing, some remarks are made about responsibility, but from the text itself it is unclear how they are not contradictory under a strict reading (“Mistakes may occur without the author having made any moral error”, p.4 and “Responsibility must be taken for any errors or omissions made” p. 6)

- 1. **France**

Title: Integrity and responsibility in research practices: a guide

Author: CNRS-CPU

Date: 2016

<URL:http://www.cpu.fr/wp-content/uploads/2016/12/integrity-and-responsibility-in-research-practices-a-guide-19.11.16.pdf>

Relevant passage on negligent: no mention of negligence.

- 1. **Germany**

Title: Guidelines for protection good scientific practice

Date: 2019

Authors: German Research Foundation (DFG)

URL: https://www.dfg.de/download/pdf/foerderung/rechtliche_rahmenbedingungen/gute_wissenschaftliche_praxis/kodex_gwp_en.pdf

Relevant passage on negligent incompetence: “Only deliberate or grossly negligent infringements defined in a set of regulations are considered scientific misconduct.” (p. 22)

- 1. **Italy**

Title: Linee guida per l’integrità nella ricerca

Date: 10 June 2015

Authors: Commissione per l’Etica della Ricerca e la Bioetica del CNR

URL: https://www.cnr.it/sites/default/files/public/media/doc_istituzionali/linee-guida-integrita-nella-ricerca-cnr-commissione_etica.pdf?v=1

Relevant passages on negligent incompetence: footnotes 22 and 23. Misconduct is stipulated as intentional, but irresponsible or questionable research practices can result from negligence. Whether or not the latter category constitutes misconduct depends on the circumstances. Under II.E., negligence can constitute misconduct; under II.B, II.D, it does not. However, II.E concerns misuse of funds and sabotage of colleagues; hence it does not concern the core activity of scientific research (but rather concerns the relationship with institutions, or with colleagues).

- 1. **Netherlands**

Title: Netherlands Code of Conduct for Research Integrity

Date: 2018

Authors: Koninklijke Nederlands Academie van Wetenschappen (KNAW), Nederlandse Federatie van Universitair Medische Centra (NFU), Nederlandse Organisatie voor Wetenschappelijk Onderzoek (NWO), TO2-Federatie, Vereniging Hogescholen, De Vereniging van Universiteiten (VSNU)

URL: <https://www.vsnu.nl/files/documents/Netherlands%20Code%20of%20Conduct%20for%20Research%20Integrity%202018.pdf>

DOI: 10.17026/dans-2cj-nvwu (Dutch version)

Relevant passage on negligent incompetence: “When it amounts to gross negligence, a questionable research practice or ‘sloppy science’ is more than a matter of mere error or carelessness but rather something that can undermine the very integrity of research.” (p. 7)

1. **Medical Profession**

- 1. **USA**

Title: AMA Code of Medical Ethics

Author: American Medical Association

Date: June 2016

URL: <https://www.ama-assn.org/sites/default/files/media-browser/principles-of-medical-ethics.pdf>

Relevant passages: principles I and II

Principle I: “A physician shall be dedicated to providing competent medical care, with compassion and respect for human dignity and rights.”

Principle II “A physician shall uphold the standards of professionalism, be honest in all professional interactions, and strive to report physicians deficient in character or competence, or engaging in fraud or deception, to appropriate entities. “

- 1. **Canada**

Title: CMA Code of Ethics

Author: Canadian Medical Association

Date: 2004

URL: <https://policybase.cma.ca/documents/policypdf/PD19-03.pdf>

Fundamental responsibility 4, “Commitment to professional integrity and competence”:

“Practise medicine competently, safely, and with integrity; avoid any influence that could undermine your professional integrity.

Develop and advance your professional knowledge, skills, and competencies through lifelong learning.”

Fundamental responsibility 5, “Commitment to professional excellence”

“Participate in establishing and maintaining professional standards and engage in processes that support the institutions involved in the regulation of the profession.”

- 1. **Australia**

Title: AMA Code of Ethics

Author: Australian Medical Association

Date: 2016

URL: <https://ama.com.au/system/tdf/documents/AMA%20Code%20of%20Ethics%202004.%20Editorially%20Revised%202006.%20Revised%202016.pdf?file=1&type=node&id=46014>

Relevant passage: section 3.1 (esp. 3.1.1, 3.1.5, 3.1.7), 3.3.1

3.1.1 Practise medicine to the best of your ability, recognising and working within your ability and scope of practice.

3.1.5 Continue lifelong professional development to keep your knowledge, skills and performance up- to-date and improve your standard of medical care.
3.1.7 Accept responsibility for maintaining and improving the standards of the profession.

3.3.1 Recognise your professional limitations and be prepared to refer as appropriate.

- 1. **UK**

Title: Good Medical Practice

Author: General Medical Council

Date: April 29, 2014

URL: https://www.gmc-uk.org/-/media/documents/good-medical-practice---english-20200128_pdf-51527435.pdf?la=en&hash=DA1263358CCA88F298785FE2BD7610EB4EE9A530

Relevant passages:

Domain 1 :

Provide a good standard of practice and care.

● Keep your professional knowledge and skills up to date.

● Recognise and work within the limits of your competence.

Principles 7, 8, 9, and 14:

Principle 7. You must be competent in all aspects of your work, including management, research and teaching.

Principle 8. You must keep your professional knowledge and skills up to date.

Principle 9. You must regularly take part in activities that maintain and develop your competence and performance.

Principle 14. You must recognise and work within the limits of your competence.”

- 1. **Europe**

Title: European Charter of Medical Ethics

Author: European Council of Medical Orders

Date: June 10, 2011

URL: <http://www.ceom-ecmo.eu/sites/default/files/documents/en-european_medical_ethics_charter-adopted_in_kos.pdf>

Relevant passage: Principle 6

Principle 6

The physician uses his professional knowledge to improve or maintain the health of those confiding in him, at their request; he may not act to their detriment under any circumstances

- 1. **Belgium**

Title: Code de Déontologie Medicale/Code van Geneeskundige Plichtenleer (Code of Medical Deontology)

Author: Conseil National de l’Ordre des Medecins/Nationale Raad van de Orde der Artsen

Date: May 25, 2020

<URL:https://www.ordomedic.be/ethics/pdf/download/fr/?XMLHttpRequest=1>

Main passages relevant to negligence: Article 3, Article 35

Article 3

"[…] whatever the branch of medical art he practices, the doctor must be fully qualified and always remain respectful of the human person.”^[[1]](#footnote-1)^

Article 35 ^[[2]](#footnote-2)^:

“Except in cases of force majeure, the doctor cannot exercise his profession under conditions that could compromise the quality of care and medical procedures.

1. Except the emergency, the doctor cannot take on a number patients such that he could not provide each of them with attentive, conscientious and respectful care.

b. The doctor cannot exceed his competence. He must seek the advice of colleagues, in particular that of specialists, either on his own initiative or at the request of the patient, whenever this appears necessary or useful in the diagnostic or therapeutic context.

Other articles that are relevant (but less directly so) for negligence: 13, 15, 21, 22, 32, 34, 36, 50, 51, 52, 89, 90, 91, 92, 93, 94, 96, 97, 98, 100, 104, 112, 126, 141, 142, 182

- 1. **France**

Title: Code de la santé publique (Code of public health)

Date: August 6, 2018

URL: <https://www.legifrance.gouv.fr/affichCode.do;jsessionid=3CEAC1EECB3D7675C4DE0E354F87663B.tplgfr41s_3?cidTexte=LEGITEXT000006072665&dateTexte=20180529>

Main passages relevant to negligence: article R4127-11

- 1. **Germany**

Title: Professional Code for Physicians in Germany

Author: German Medical Assembly

Date: 2011

URL: https://www.bundesaerztekammer.de/fileadmin/user_upload/downloads/MBOen2012.pdf

Main passages relevant to negligence: Article 4

Article 2:

[…] “Conscientious practise of the profession requires, in particular, the necessary professional qualification and compliance with the accepted state of medical knowledge.”

Article 4:

[…] Physicians who practise their profession are obliged to engage in continuing medical education to the extent necessary to maintain and develop the competence required in practising their profession.

- 1. **Italy**

Title: Codice di deontologia medica (Code of medical deontology)

Author: Federazione Nazionale degli Ordini dei Medici Chirurghi e degli Odontoiatri

Date: 2014

URL: <https://www.studiocataldi.it/normativa/codice-deontologia-medica/>

Main passages relevant to negligence: Article 3 and article 19.

Article 3^[[3]](#footnote-3)^ :

In order to protect individual and collective health, the doctor carries out activities based on the specific and exclusive competences set out in the educational objectives of the Degree Courses in Medicine and Surgery and Dentistry and Dental Prosthetics, integrated and expanded by the development of knowledge in medicine, technical and non-technical skills related to professional practice, organizational and managerial innovations in healthcare, teaching and research.

Article 19:

The doctor, throughout his professional life, pursues constant updating and continuous training for the development of technical and non-technical professional knowledge and skills, favoring their diffusion to learners and collaborators.^[[4]](#footnote-4)^

- 1. **Netherlands**

Title: Gedragsregels voor artsen

Author: Koninklijke Nederlandsche Maatschappij tot bevordering der Geneeskunst (KNMG)

Date: 2018

URL: <https://www.knmg.nl/advies-richtlijnen/knmg-publicaties/gedragsregels-voor-artsen-2.htm>

Main passages relevant to negligence: I.3

Article I. 3^[[5]](#footnote-5)^

The care provided by the doctor must be of good quality. Relevant aspects in this context are: expertise; effectiveness and efficiency; patient orientation; carefulness; safety. The doctor retains his medical knowledge and skills from that part of the medicine that he practices level and where possible contribute to its development. Further and further training are hereby a necessity.

1. Our translation. Original text :

   […] le médecin doit, quelle que soit la branche de l'art médical qu'il pratique, être pleinement qualifié et demeurer toujours respectueux de la personne humaine. [↑](#footnote-ref-1)
2. Our translation. Original text :

   Sauf cas de force majeure, le médecin ne peut exercer sa profession dans des conditions qui pourraient compromettre la qualité des soins et des actes médicaux.

   a. Hors l'urgence, le médecin ne peut prendre en charge un nombre de patients tel qu'il ne pourrait assurer à chacun d'entre eux des soins attentifs, consciencieux et respectueux de la personne humaine

   b. Le médecin ne peut outrepasser sa compétence. Il doit prendre l'avis de confrères, notamment de spécialistes, soit de sa propre initiative, soit à la demande du patient, chaque fois que cela paraît nécessaire ou utile dans le contexte diagnostique ou thérapeutique. [↑](#footnote-ref-2)
3. Our translation. Original text :  « Al fine di tutelare la salute individuale e collettiva, il medico esercita attività basate sulle competenze, specifiche ed esclusive, previste negli obiettivi formativi degli Ordinamenti didattici dei Corsi di Laurea in Medicina e Chirurgia e Odontoiatria e Protesi dentaria, integrate e ampliate dallo sviluppo delle conoscenze in medicina, delle abilità tecniche e non tecniche connesse alla pratica professionale, delle innovazioni organizzative e gestionali in sanità, dell'insegnamento e della ricerca. » [↑](#footnote-ref-3)
4. Our translation. Original text : « Il medico, nel corso di tutta la sua vita professionale, persegue l'aggiornamento costante e la formazione continua per lo sviluppo delle conoscenze e delle competenze professionali tecniche e non tecniche, favorendone la diffusione ai discenti e ai collaboratori. » [↑](#footnote-ref-4)
5. Our translation. Original text: “De hulpverlening door de arts dient van goede kwaliteit te zijn. Relevante aspecten in dat verband zijn: deskundigheid; doeltreffendheid en doelmatigheid ; patiëntgerichtheid; zorgvuldigheid; veiligheid. De arts houdt zijn medische kennis en vaardigheden van dat deel van de geneeskunst dat hij beoefent op peil en levert waar mogelijk aan de ontwikkeling daarvan een bijdrage. Na- en bijscholing zijn hierbij noodzaak.” [↑](#footnote-ref-5)
